# Brazil-Portugal Transcultural Adaptation of the UWES-9: Internal Consistency, Dimensionality, and Measurement Invariance

**DOI:** 10.3389/fpsyg.2018.00353

**Published:** 2018-03-20

**Authors:** Jorge Sinval, Sonia Pasian, Cristina Queirós, João Marôco

**Affiliations:** ^1^Faculty of Philosophy, Sciences and Letters of Ribeirão Preto, University of São Paulo, São Paulo, Brazil; ^2^Faculty of Psychology and Education Sciences, University of Porto, Porto, Portugal; ^3^William James Centre for Research, ISPA-Instituto Universitário, Lisboa, Portugal

**Keywords:** work engagement, Portuguese-Brazilian version, measurement invariance, reliability of the scores, validity evidence, psychometric properties

## Abstract

The aim of this paper is to present a revision of international versions of the Utrecht Work Engagement Scale and to describe the psychometric properties of a Portuguese version of the UWES-9 developed simultaneously for Brazil and Portugal, the validity evidence related with the internal structure, namely, Dimensionality, measurement invariance between Brazil and Portugal, and Reliability of the scores. This is the first UWES version developed simultaneously for both countries, and it is an important instrument for understanding employees' work engagement in the organizations, allowing human resources departments to better use workforces, especially when they are migrants. A total of 524 Brazilian workers and 522 Portuguese workers participated in the study. Confirmatory Factor Analysis, group comparisons, and Reliability estimates were used. The use of workers who were primarily professionals or administrative support, according to ISCO-08, reinforced the need to collect data on other professional occupations. Confirmatory factor analysis showed acceptable fit for the UWES-9 original three-factor solution, and a second-order factor structure has been proposed that presented an acceptable fit. Full-scale invariance was obtained between the Portuguese and Brazilian samples, both for the original three-factor first-order and second-order models. Data revealed that Portuguese and Brazilian workers didn't show statistically significant differences in the work engagement dimensions. This version allows for direct comparisons of means and, consequently, for performance of comparative and cross-cultural studies between these two countries.

## Introduction

The global economic crisis elicited important changes in the workforce (The Organisation for Economic Co-operation and Development; Pilati et al., 2015; OECD, 2016), namely workers changing their physical location in order to have a better salary or even a job. International enterprises also frequently changed their physical location to decrease taxes, for political reasons or to achieve lower labor costs. Moreover, workers' career expectations increased occupational mobility (Dunkerley, 2013). These factors contributed to a growing flow of international workers (Dollard et al., 2014). Due to the commonalities in language and cultural values, Brazilians are the majority of foreign workers in Portugal, and vice-versa (INE, 2014). In 2014, Portuguese workers represented 1% of European labor inflows while Brazilians represented 1.1% (OECD, 2016). The Brazilian migrants dominate the inflow entries to Portugal at 21% (Serviço de Estrangeiros e Fronteiras, 2016). Reciprocal migrant flows created new challenges to enterprises' functioning and in research about occupational health, cross-cultural studies and bias in personality assessment (van de Vijver, 2000; Hofstede et al., 2010). Moreover, Occupational Health departments are continuously alert for the psychosocial risks at work and workers' well-being, referring to positive mental health as a promoter of active and healthy aging (Direção-Geral da saúde, 2013; European Agency for Safety Health at Work, 2016).

Simultaneously, psychology reinforces the need to research new challenges instead of negative topics and to valorize well-being at work (Scorsolini-Comin et al., 2013; van Veldhoven and Peccei, 2015; Mäkikangas et al., 2016) or positive states such as engagement within Positive Occupational Psychology (Bakker et al., 2008, 2012; Byrne, 2014; Truss et al., 2014; Chambel, 2016; Salanova et al., 2016). Since engaged workers seem to be more energetic, enthusiastic regarding their work and have greater initiative, innovativeness and well-being (Alfes et al., 2013; Shuck et al., 2014; Bal and De Lange, 2015; de Camargo et al., 2015; Sattar et al., 2015; Graffigna, 2017; Presbitero, 2017), it is well documented that regarding human resources, work engagement plays a mediating role between organizations' human resources management practices and workers' job satisfaction, performance outcomes, team cohesion, reduced turnover or reduced job stress.

Work engagement can be defined as a positive motivational construct characterized by vigor, dedication, and absorption and, within workplaces' psychology, it is generally seen as a protector against burnout (Schaufeli and Salanova, 2007; Schaufeli and Bakker, 2010). Vigor refers to high levels of persistence, energy, and mental resilience while working, and the willingness to invest effort in one's work. Dedication is characterized by being strongly involved in one's work and experiencing a sense of meaning, enthusiasm, inspiration, and pride. Finally, absorption refers to being completely concentrated and happily immersed in one's work, with distortion of time and intrinsic enjoyment (Schaufeli et al., 2002b). Globally, work engagement is referred to a cognitive-affective persistent state in time, which is not focused on an object or a specific behavior (Salanova et al., 2000; Schaufeli, 2017). It is related to performance (Pittenger, 2015; Knight et al., 2017; Reijseger et al., 2017) and to economy, governance, and culture (Schaufeli, 2017). A recent report presented by Schaufeli (2017) analyses data from 43,850 employees from thirty-five European countries, from the “*6th European Working Conditions Survey–*2015,” aiming to identify the relationships between work engagement at the country level and several national economic, governance and cultural indicators. The author concludes that the most engaged countries are in Northwestern Europe, whereas the least engaged are located in Southern Europe or the Balkans, with Portugal being under the European average (3.69 vs. 3.94 on a 1–5 scale).

The most disseminated instrument to measure work engagement is the Utrecht Work Engagement Scale—UWES (Mäkikangas et al., 2016; Knight et al., 2017), created by Schaufeli and Bakker (2003). Its properties need to be investigated, to use it with more rigor. This paper aims to present a revision of international versions of the Utrecht Work Engagement Scale and to describe psychometric properties of a Portuguese version of the UWES-9 developed simultaneously for Brazil and Portugal. The UWES has a version with 17 items, distributed among three dimensions as follows: vigor (6 items), dedication (5 items), and absorption (6 items). Despite a very recent and ultra-short measure of work engagement with 3 items (Schaufeli et al., 2017), a short version is frequently used having 9 items (UWES-9), 3 for each dimension, and is recommended by some authors over the longer version (Nerstad et al., 2010; Hernandez-Vargas et al., 2016), in order to improve data quality, with advantages such as the potential decrease of missing values, the shorter amount of time to complete the questionnaire, and the increased likelihood of completing the questionnaire (Kruyen et al., 2013). This short version produced data with good psychometric properties when it was developed in the original sample (Schaufeli et al., 2006), but also in other recent independent studies (de Bruin and Henn, 2013; Littman-Ovadia and Balducci, 2013; Panthee et al., 2014; Fong and Ho, 2015; Vazquez et al., 2015). The internal consistency (Cronbach's Alpha) usually shows acceptable values (Fong and Ng, 2012) and, although is common to find high inter-factor correlations between the three dimensions, the factorial invariance of the UWES across countries has been demonstrated in student samples (Schaufeli et al., 2002a). Balducci et al. (2010) used a sample of Italian and Dutch workers, where they applied each country's version, instead of the same transcultural version, due to language specificities. Also in Greek and Dutch employees, measurement invariance was obtained using each national language (Xanthopoulou et al., 2012). Schaufeli et al. (2006) selected items through an iterative process from the original scale to rebuild the UWES-9 and obtained a version with good psychometric proprieties, namely Cronbach's α higher than 0.80 and a three-factor solution that fit the data better than a one-factor solution.

Being a recent and positive occupational measure, and due to its ease of use, the UWES has been translated into many languages (Schaufeli and Bakker, 2010), such as Serbian (Petrović et al., 2017), Spanish (Schaufeli et al., 2002a; Hernandez-Vargas et al., 2016), Hebrew (Littman-Ovadia and Balducci, 2013), Chinese (Fong and Ng, 2012), Norwegian (Nerstad et al., 2010), Italian (Balducci et al., 2010), Japanese (Shimazu et al., 2008), and Portuguese (Schaufeli and Bakker, 2003; Vazquez et al., 2015), showing the robustness and relevance of the three-dimensional construct of work engagement among different cultures. Despite the recent literature review of Kulikowski (2017a), in Table 1 we present a revision of some adapted versions based on the UWES-17 version or on reduced versions, using as criteria the UWES' application to workers in different countries, as well psychometric properties, considering not only confirmatory analysis referred by Kulikowski but also Exploratory Factor Analysis. Thus, we included more studies, allowing a more global perspective on UWES versions. The UWES has also been used among different occupational groups (e.g., students, hospital staff, managers, police officers, teachers), and a study by Seppälä et al. (2009) showed that the UWES-9's structure, but not the UWES-17's structure, remained relatively unchanged among the different occupational groups. Extremera et al. (2012) found factorial invariance for the Spanish UWES-15 between genders in a multi-occupational sample, and between two different multi-occupational samples. More recently, Rodríguez-Montalbán et al. (2014) concluded that the UWES-9 has a better fit to a tri-factorial structure, while Hernandez-Vargas et al. (2016) confirmed the three-factorial structure in the UWES-9 and UWES-17 scales, concluding that the UWES-9 showed a better goodness-of-fit and measurement invariance among two Mexican samples.

**Table 1:** UWES versions and its Goodness-of-fit details.

**Country (Authors)**	**Occupational group**	***N***	**Items version (factors)**	**Total α**	**χ^2^/*df***	***TLI/NNFI***	***GFI***	***CFI***	***RMSEA***	***SRMR***
Portugal (Sinval et al., 2018)	Rescue Workers	3,887	CFA 17 (three)	0.96	27.30	0.993	–	0.994	0.091	–
			CFA 9 (three)	0.95	40.91	0.995	–	0.997	0.105	–
			CFA 9 (2nd order-three)		20.76	0.997	–	0.998	0.074	–
India	Information Technology	100	PCA 17 (one)	0.93	–	–	–	–	–	–
Thailand		100		0.92	–	–	–	–	–	–
(Chaudhary et al., 2018)		(200)		0.93	–	–	–	–	–	–
Belgium	Various	5,062	CFA 3 (one)	0.85	–	–	–	–	–	–
			CFA 9 (three)	0.93	–	–	–	–	–	–
Finland		22,117	CFA 3 (one)	0.80	–	–	–	–	–	–
			CFA 9 (three)	0.94	–	–	–	–	–	–
Japan		1,968	CFA 3 (one)	0.85	–	–	–	–	–	–
			CFA 9 (three)	0.95	–	–	–	–	–	–
Netherlands		38,278	CFA 3 (one)	0.82	–	–	–	–	–	–
			CFA 9 (three)	0.94	–	–	–	–	–	–
Spain		10,040	CFA 3 (one)	0.77	–	–	–	–	–	–
			CFA 9 (three)	0.90	–	–	–	–	–	–
(Schaufeli et al., 2017)		(77,465)	–	–	–	–	–	–	–	–
India (Lathabhavan et al., 2017)	Bank	467	CFA 17 (one)	–	7.48	0.86	0.82	0.91	0.13	0.12
			CFA 17 (three)		6.07	0.92	0.83	0.93	0.10	0.03
			CFA 15 (one)	–	6.83	0.77	0.86	0.93	0.10	0.11
			CFA 15 (three)		5.67	0.83	0.88	0.94	0.10	0.03
			CFA 9 (one)	–	5.38	0.87	0.94	0.96	0.10	0.10
			CFA 9 (three)		1.91	0.96	0.98	0.99	0.04	0.03
Iran (Torabinia et al., 2017)	Nurses	282	EFA 17 (three)	0.84	–	–	–	–	–	–
Malaysia (Yew et al., 2017)	Primary School Teachers	345	CFA 17 (three)	–	–	0.921	–	0.933	0.084	0.032
Poland (Kulikowski, 2017b)	Various	1,420	CFA 17 (three)	–	21.53	–	–	0.84	0.12	–
			CFA 17 (two)		22.56	–	–	0.83	0.12	–
			CFA 17 (one)		25.37	–	–	0.82	0.13	–
			CFA 15 (three)	–	21.75	–	–	0.85	0.12	–
			CFA 15 (two)		23.65	–	–	0.84	0.13	–
			CFA 15 (one)		25.23	–	–	0.82	0.13	–
			CFA 9 (three)	0.90	20.72	–	–	0.94	0.12	–
			CFA 9 (two)		32.68	–	–	0.89	0.15	–
			CFA 9 (one)		33.60	–	–	0.88	0.15	–
			CFA 6 (two)	0.87	15.78	–	–	0.97	0.10	–
			CFA 6 (one)		35.44	–	–	0.93	0.16	–
Russia (Lovakov et al., 2017)	Energy	1,783	CFA 9 (one)	–	23.35	0.87	–	0.90	0.11	0.05
			CFA 9 (three)	–	25.70	0.85	–	0.90	0.12	0.05
Serbia (Petrović et al., 2017)	Various	860	EFA 17 (three)	0.92	–	–	–	–	–	–
			CFA 17 (one)		3.91	–	0.920	0.695	0.062	0.079
			CFA 17 (three)		3.63	–	0.928	0.732	0.056	0.077
			CFA 17 (three - from EFA)		3.58	–	0.929	0.736	0.055	0.070
			CFA 9 (one)	0.90	5.37	–	0.951	0.832	0.072	0.083
			CFA 9 (three)		4.86	–	0.960	0.868	0.067	0.100
Sierra Leone (Vallières et al., 2017)	Community Health Workers	334	CFA 17 (one)	–	2.23	0.689	–	0.728	0.062	0.069
			CFA 17 (bi-factor)		1.92	0.767	–	0.825	0.054	0.057
			CFA 9 (one)	–	1.82	0.882	–	0.911	0.050	0.050
			CFA 9 (three)		2.01	0.854	–	0.902	0.056	0.056
			CFA 9 (bi-factor)		2.14	0.844	–	0.921	0.059	0.059
South Korea (Ho Kim et al., 2017)	White-collar	307	EFA 9 (one)	–	29.58	0.577	–	0.683	0.239	0.154
			EFA 9 (two - VI, DE+AB)		6.8	0.860	–	0.926	0.137	0.029
			EFA 9 (three)		2.9	0.954	–	0.985	0.079	0.013
		342	CFA 9 (one)	–	13.93	0.770	–	0.827	0.194	0.053
			CFA 9 (two - VI, DE+AB)		6.99	0.893	–	0.923	0.132	0.044
			CFA 9 (three)		3.86	0.949	–	0.966	0.091	0.035
		(649)	–	–	–	–	–	–	–	–
Germany	German workers in Germany	1,406	4 (one)	0.89	–	–	–	–	–	–
	Turkish workers in Germany	201		0.90	–	–	–	–	–	–
Turkey	Turkish workers in Turkey	362		0.89	–	–	–	–	–	–
(Ulusoy et al., 2016)	(Multi-occupational)	(1,969)		0.89	–	–	–	–	–	–
Malaysia (Sulaiman and Zahoni, 2016)	Salespersons	205	EFA 14 (two)	0.51	–	–	–	–	–	–
México (Hernandez-Vargas et al., 2016)	Health	475	CFA 15 (one)	–	5.19	0.66	–	0.71	0.09	–
			CFA 15 (three)		2.53	0.88	–	0.90	0.05	–
			CFA 9 (one)	–	8.50	0.64	–	0.93	0.13	–
			CFA 9 (three)		1.70	0.97	–	0.98	0.04	–
Saudi Arabia (Eman-Nafa, 2016)	Primary School Teachers	414	9 (three)	0.85	–	–	–	–	–	–
Switzerland (Zecca et al., 2015)	Multi-occupational	661	CFA 17 (one)	0.93	4.04	0.80	–	0.82	0.12	0.07
			CFA 17 (three)		3.05	0.86	–	0.88	0.10	0.08
			CFA 9 (one)	0.92	21.33	0.82	–	0.87	0.18	0.06
			CFA 9 (three)		15.44	0.89	–	0.93	0.14	0.08
United States of America (Matz-Costa, 2016)	Various	2,195	9 (three)	0.91	–	–	–	–	–	–
Peru (Flores Jiménez et al., 2015)	Teachers	145	CFA 15 (one)	–	2.65	–	–	0.871	0.107	0.105
			CFA 15 (two)		2.16	–	–	0.911	0.090	0.085
			CFA 15 (three)		1.96	–	–	0.928	0.082	0.073
			CFA 9 (one)	–	1.88	–	–	0.956	0.078	0.092
			CFA 9 (two)		1.12	–	–	0.999	0.029	0.064
			CFA 9 (three)		0.76	–	–	1.000	0.000	0.042
Brazil (Vazquez et al., 2015)	Various	1,167	CFA 17 (one)	0.95	15.16	0.95	–	0.96	0.10	–
			CFA 17 (three)		13.67	0.95	–	0.96	0.10	–
			CFA 9 (one)	0.94	10.05	0.97	–	0.98	0.13	–
			CFA 9 (three)		7.16	0.98	–	0.98	0.12	–
Germany (Sautier et al., 2015)	Patients with	179	CFA 9 (one)	0.94	5.48	0.88	0.85	0.91	0.16	–
	hematological		CFA 9 (three)		5.82	0.87	0.86	0.91	0.17	–
	malignancies									
China (Fong and Ho, 2015)	Health	1,112	CFA 9 (one)	–	4.88	–	–	0.903	0.084	–
			CFA 9 (three)		3.12	–	–	0.936	0.072	–
			CFA 9 (partial bi-factor)		2.59	–	–	0.941	0.074	–
			BCFA 9 (one)		–	–	–	–	–	–
			BCFA 9 (three)		–	–	–	–	–	–
			BCFA 9 (partial bi-factor)		–	–	–	–	–	–
Italy (Villotti et al., 2014)	Mental ill workers	310	CFA 9 (one)	0.94	5.45	0.956	–	0.967	0.132	–
			CFA 9 (three)		3.15	0.979	–	0.986	0.092	–
Mexico (Villavicencio-Ayub et al., 2014)	Various	120	EFA 17 (three)	0.90	–	–	–	–	–	–
		904	CFA 17 (three from EFA)		5.42	0.96	–	0.97	0.07	–
		(1024)	–	–	–	–	–	–	–	–
Nepal (Panthee et al., 2014)	Nurses	438	CFA 17 (one)	0.72	3.72	0.86	0.88	0.88	0.07	–
			CFA 17 (three)		3.43	0.87	0.89	0.89	0.07	–
			CFA 9 (one)	0.76	5.35	0.89	0.92	0.92	0.10	–
			CFA 9 (two)		4.50	0.91	0.94	0.93	0.08	–
			CFA 9 (three)		3.75	0.93	0.95	0.95	0.07	–
Puerto Rico	Various	2,796	CFA 9 (one)	0.92	25.82	0.91	–	0.92	–	0.04
(Rodríguez-Montalbán et al., 2014)			CFA 9 (three)		19.46	0.93	–	0.95	–	0.03
Pakistan (Yusoff et al., 2013)	Academic staff	400	CFA 9 (one)	0.87	5.57	–	0.69	0.78	0.126	–
			CFA 9 (two)		4.96	–	0.78	0.88	0.164	–
			CFA 9 (three)		2.81	–	0.98	0.99	0.064	–
Italy (Simbula et al., 2013)	Teachers	488	CFA 17 (one)	–	6.77	0.84	0.82	0.86	0.11	–
			CFA 17 (three)		5.76	0.87	0.84	0.89	0.10	–
			CFA 9 (one)	–	9.16	0.89	0.89	0.91	0.13	–
			CFA 9 (three)		7.45	0.91	0.92	0.94	0.11	–
Israel (Littman-Ovadia and Balducci, 2013)	White-collar	252	CFA 9 (one)	0.93	4.23	–	–	0.98	0.11	0.04
			CFA 9 (three)		2.81	–	–	0.99	0.08	0.04
Chile (Gilchrist et al., 2013)	Health	165	EFA 17 (two)	0.87	–	–	–	–	–	–
South Africa (de Bruin and Henn, 2013)	Various	369	CFA 9 (one)	–	6.95	0.958	–	0.943	0.128	–
			CFA 9 (three)		5.12	0.974	–	0.961	0.107	–
			CFA 9 (partial bi-factor)		1.69	0.996	–	0.993	0.044	–
India (Alok, 2013)	Professionals	182	CFA 9 (one)	0.85	3.40	–	–	0.893	0.115	0.063
			CFA 9 (two)		3.52	–	–	0.892	0.118	0.063
			CFA 9 (2nd order - two)		3.52	–	–	0.892	0.118	0.063
United States of America (Mills et al., 2012)	County extension agents	98	CFA 17 (one)	0.91	2.18	–	–	0.83	0.11	–
			CFA 17 (two - DE, VI +AB)		2.14	–	–	0.83	0.11	–
			CFA 17 (three)		2.98	–	–	0.71	0.11	–
			CFA 9 (one)	0.90	2.41	–	–	0.92	0.12	–
			CFA 9 (two - DE, VI +AB)		2.29	–	–	0.93	0.12	–
			CFA 9 (three)		1.93	–	–	0.95	0.10	–
	Various	120	CFA 17 (one)	0.89	2.50	–	–	0.81	0.11	–
			CFA 17 (two - DE, VI +AB)		2.48	–	–	0.81	0.11	–
			CFA 17 (three)		2.47	–	–	0.81	0.11	–
			CFA 9 (one)	0.84	2.07	–	–	0.94	0.09	–
			CFA 9 (two - DE, VI +AB)		2.57	–	–	0.91	0.11	–
			CFA 9 (three)		2.74	–	–	0.90	0.12	–
		(218)	–	–	–	–	–	–	–	–
United States of America (Wefald et al., 2012)	Financial	382	CFA 9 (one)	0.93	12.65	–	0.83	0.87	0.18	–
			CFA 9 (three)		10.42	–	0.88	0.91	0.16	–
New Zealand (Viljevac et al., 2012)	Call centers	139	CFA 17 (three)	–	1.95	0.878	–	0.905	0.083	–
Hong Kong (Fong and Ng, 2012)	Elderly service	992	CFA 17 (one)	–	7.66	0.80	–	0.83	0.08	0.07
			CFA 17 (three)		7.37	0.81	–	0.84	0.08	0.07
			CFA 15 (one)	–	7.96	0.81	–	0.84	0.08	0.07
			CFA 15 (three)		8.05	0.81	–	0.84	0.09	0.07
			CFA 9 (one)	0.88	8.51	0.87	–	0.90	0.09	0.05
			CFA 9 (three)		7.18	0.90	–	0.93	0.08	0.05
Greece	Various	206	CFA 15 (one)	–	3.71	–	0.80	0.89	0.12	0.05
			CFA 15 (two - VI, DE+AB)		3.52	–	0.82	0.90	0.11	0.05
			CFA 15 (two - DE, VI+AB)		3.55	–	0.83	0.90	0.11	0.05
			CFA 15 (two - AB, VI+DE)		3.72	–	0.80	0.89	0.12	0.05
			CFA 15 (three)		3.43	–	0.83	0.91	0.11	0.05
Netherlands		162	CFA 15 (one)	–	3.34	–	0.79	0.88	0.12	0.06
			CFA 15 (two - VI, DE+AB)		3.12	–	0.80	0.89	0.12	0.07
			CFA 15 (two - DE, VI+AB)		2.97	–	0.81	0.90	0.11	0.06
			CFA 15 (two - AB, VI+DE)		3.24	–	0.80	0.88	0.12	0.06
			CFA 15 (three)		2.93	–	0.82	0.90	0.11	0.06
(Xanthopoulou et al., 2012)		(368)	MGCFA 15 (three)	–	3.18	–	–	0.90	0.08	0.05
Argentina (Spontón et al., 2012)	Various	337	EFA 16 (two)	0.89	–	–	–	–	–	–
		337	CFA 17 (one)	0.90	3.17	0.88	0.88	0.89	0.081	–
			CFA 17 (three)		2.93	0.89	0.89	0.91	0.077	–
			CFA 16 (two - from EFA)	0.89	2.93	0.90	0.89	0.91	0.077	–
		(674)	–	–	–	–	–	–	–	–
Australia	Teachers	208	CFA 9 (one)	0.93	8.77	–	–	0.86	0.19	–
			CFA 9 (three)		6.96	–	–	0.90	0.17	–
Canada		256	CFA 9 (one)	0.88	9.47	–	–	0.80	0.18	–
			CFA 9 (three)		5.04	–	–	0.92	0.13	–
Hong Kong		100	CFA 9 (one)	0.93	4.05	–	–	0.88	0.18	–
			CFA 9 (three)		2.58	–	–	0.95	0.13	–
Indonesia		100	CFA 9 (one)	0.81	2.79	–	–	0.86	0.13	–
			CFA 9 (three)		2.97	–	–	0.87	0.14	–
Oman		192	CFA 9 (one)	0.90	5.91	–	–	0.86	0.16	–
			CFA 9 (three)		4.72	–	–	0.91	0.14	–
(Klassen et al., 2012)		(856)	CFA 9 (one)	0.93	8.77	–	–	0.86	0.19	–
India (Chaudhary et al., 2012)	Manufacturing and Services	438	CFA 17 (one)	0.90	2.59	0.905	0.921	0.917	0.060	–
			CFA 17 (three)		2.30	0.922	0.933	0.933	0.055	–
			CFA 9 (one)	0.82	2.34	0.962	0.970	0.962	0.055	–
			CFA 9 (three)		2.42	0.964	0.972	0.964	0.057	–
Netherlands (Breevaart et al., 2012)	Various	271	MGCFA 9 (one)	–	14.66	–	–	0.90	0.10	0.05
			MGCFA 9 (three)		6.61	–	–	0.96	0.06	0.04
Italy	White-collar	668	CFA 9 (one)	–	21.71	0.710	0.760	0.780	0.230	–
			CFA 9 (three)		7.20	0.894	0.898	0.929	0.137	–
			CFA 9 (three - Mod.)		2.93	0.967	0.961	0.982	0.077	–
Netherlands		2,213	CFA 9 (one)	–	14.81	0.913	0.916	0.935	0.112	–
			CFA 9 (three)		8.96	0.950	0.959	0.967	0.085	–
			CFA 9 (three - Mod.)		7.97	0.956	0.967	0.973	0.080	–
(Balducci et al., 2010)		(2,881)	MGCFA 9 (three)	0.92	5.57	0.958	0.965	0.975	0.057	–
Norway (Nerstad et al., 2010)	Various	1,266	CFA 17 (one)	–	17.52	–	–	0.96	0.11	–
			CFA 17 (two)		13.04	–	–	0.97	0.10	–
			CFA 17 (three)		12.26	–	–	0.97	0.09	–
			CFA 9 (one)	–	24.91	–	–	0.95	0.14	–
			CFA 9 (two)		7.01	–	–	0.99	0.07	–
			CFA 9 (three)		7.43	–	–	0.99	0.07	–
Finland (Seppälä et al., 2009)	Various	9,404	CFA 17 (three)	–	7.83	0.95	–	0.96	0.064	–
			CFA 9 (three)	–	11.07	0.96	–	0.98	0.076	–
USA (Muilenburg-Trevino, 2009)	Non-profit Organization Employees	227	EFA 15 (one)	0.94	–	–	–	–	–	–
			CFA 17 (one)	0.93	5.04	–	0.74	0.94	0.14	–
			CFA 17 (three)		3.36	–	0.82	0.96	0.11	–
Japan (Shimazu et al., 2008)	Engineers and Nurses	2,334	CFA 17 (one)	–	30.52	–	0.81	0.85	0.11	–
			CFA 9 (one)	0.92	12.86	–	0.90	0.92	0.07	–
Australia	Various	473	–	–	–	–	–	–	–	–
Belgium		767	–	–	–	–	–	–	–	–
Canada		267	–	–	–	–	–	–	–	–
Finland		3,651	–	–	–	–	–	–	–	–
France		221	–	–	–	–	–	–	–	–
Germany		465	–	–	–	–	–	–	–	–
Netherlands		2,163	–	–	–	–	–	–	–	–
Norway		2,114	–	–	–	–	–	–	–	–
South Africa		2,547	–	–	–	–	–	–	–	–
Spain		1,832	–	–	–	–	–	–	–	–
(Schaufeli et al., 2006)		(14,521)	CFA 9 (one)	0.80	22.76	0.89	0.89	0.91	0.04	–
			CFA 9 (three)		13.45	0.93	0.95	0.96	0.03	–
Sweden (Hallberg and Schaufeli, 2006)	Information	186	CFA 9 (one)	0.93	4.12	0.95	–	0.97	0.13	0.05
	Communication		CFA 9 (three)		3.91	0.96	–	0.97	0.13	0.04
	Technology consultants									
China (Yi-wen and Yi-qun, 2005)	Middle School Teachers	277	CFA 15 (one)	0.90	2.74	–	0.89	0.89	0.081	–
			CFA 15 (three)		2.83	–	0.89	0.89	0.083	–
			CFA 15 (three - Mod.)		1.55	–	0.94	0.97	0.046	–
South Africa (Storm and Rothmann, 2003)	Police Officers	2,396	CFA 17 (three)	–	17.06	0.91	0.90	0.92	0.08	–
			CFA 17 (one)		18.91	0.90	0.87	0.91	0.09	–
			CFA 15 (three)	–	13.30	0.93	0.94	0.95	0.07	–
			CFA 13 (one)	–	12.34	0.95	0.95	0.96	0.06	–
Germany (Sonnentag, 2003)	Public Service	147	PCA 17 (one)	0.91	–	–	–	–	–	–

*The extracted results are presented with two or three decimal places depending of the original authors report. Mod., Modification Indices; BCFA, Bayesian Confirmatory Factor Analysis; CFA, Confirmatory Factor Analysis; EFA, Exploratory Factor Analysis; MGCFA, Multi-Group Confirmatory Factor Analysis; PCA, Principal Component Analysis; GFI, goodness-of-fit index; RMSEA, Root Mean Square Error of Approximation; NNFI, Non Normed Fit Index; TLI, Tucker Lewis Index; CFI, Comparative Fit Index; SRMR, Standardized Root Mean Square Residual*.

While researchers have translated the UWES' student version into Portuguese (UWES-S, version of 14-item from UWES-17) and confirmed its good psychometric properties or invariance (Schaufeli et al., 2002a; Cadime et al., 2016), there is a lack of rigorous validation studies with either version of the UWES among Portuguese workers. Some studies used the 24-item original version (e.g., Picado et al., 2008), while others used only the vigor and dedication “core” work engagement scales (e.g. Chambel, 2012), thereby jeopardizing a better understanding of work engagement among Portuguese workers. Additionally, cross-cultural studies with the UWES among students were performed in several countries, including the Philippines and Argentina (Mesurado et al., 2016), Spain and the Netherlands (Schaufeli et al., 2002a), Turkey (Çapri et al., 2017), Ecuador (Portalanza Chavarría et al., 2017), South Africa (Mostert et al., 2017), South Korea (Römer, 2016), Puerto Rico (Sánchez-Cardona et al., 2016), India (Rastogi et al., 2017), Japan (Tsubakita et al., 2017), Lithuania (Paradnike and Bandzevičiene, 2015), China (Meng and Jin, 2017), and the USA (Mills et al., 2012), looking for the cultural influences and impact of national values on participants and organizations (Hofstede et al., 2010).

To perform cross-cultural studies, including personality assessment (Dana, 2000), or to compare constructs within nations (Davidov et al., 2014), equivalent construct measurements are required, allowing meaningful comparisons of means or/and relations between constructs. The latent structure and its items should have stability across countries (measurement invariance according to van de Schoot et al., 2015). Although this seems to be a logical prerequisite, measurement invariance is rarely tested as it should be (Davidov et al., 2014), and sometimes when it is tested, it is done without adequate parametrization for ordinal items (Koh and Zumbo, 2008; Hirschfeld and von Brachel, 2014). If measurement invariance is not obtained, the constructs' meaning is different and means of each group cannot be compared (Putnick and Bornstein, 2016). Considering the existence of a Brazilian UWES version (Vazquez et al., 2015) but the absence of a Portuguese version for multi-occupational groups, and, especially, the absence of a UWES-9 version adapted simultaneously for both countries, it seems crucial to develop a unique version that will allow development of comparative studies of workers‘ work engagement in Portugal and Brazil. This version would be useful for future studies extended to other countries with Portuguese as the official language, such as the African Nations of Angola and Mozambique, Guinea-Bissau, Cape-Verde and São Tomé and Príncipe. Response bias is more likely to occur with long and time-consuming scales, while short versions tend to reduce this kind of bias. They are also easier to use, especially when they are used with other scales.

As far as we know, this is the first study using the same UWES version developed for both countries. We aim to assess the validity evidence related to the internal structure of the Portuguese version (PT-BR and PT-PT) of the original Utrecht Work Engagement Scale 9 items version (Schaufeli et al., 2006). More specifically, we evaluated: the UWES' Dimensionality, measurement invariance, and Reliability of the scores accordingly to the *Standards for Educational and Psychological Testing* framework (American Educational Research Association, 2014), and the existence of a work engagement second-order factor. We compared the fit of the original UWES-9 tri-factorial structure between Brazil and Portugal, considering cultural similarities between samples of Brazilian and Portuguese workers, and also the existence of numerous versions of UWES; we hypothesized: (H1) the original UWES-9 three-factor first-order structure will present evidence of construct validity; (H2) an invariant structure for both countries; (H3) and will show a second-order latent factor, *work engagement*, with validity evidence that supports its usage among workers from Portugal and Brazil; (H4) the UWES-9 second-order latent factor model will show measurement invariance between countries; and, (H5) UWES-9's dimensions will present different mean scores for Brazilian and Portuguese workers due to socio-cultural differences between the two countries.

## Method

### Participants

A sample of 524 Brazilian workers and a sample of 522 Portuguese workers completed the UWES-9. Participation was anonymous and voluntary. The average age was 35.57 years old (*SD* = 10.04), with 64% being female. Using the *International Standard Classification of Occupations*- *ISCO-08* (International Labour Office, 2012) to allow for comparisons between countries, worker's occupations were mainly categorized as professional or administrative support (Table 2). Regarding educational level, 78% of the sample had, at the least, graduated from college. The high percentage of Masters in Portugal is due to recent changes in graduation, corresponding mostly to integrated masters, which are similar to college graduation in Brazil. We used non-probabilistic convenience sampling, with the following inclusion criteria: all participants had, as workers, a contract or formal ties with their employers, were able to read, and had easy access to a PC, smartphone or tablet, to access the online platform where the instruments were deployed.

**Table 2:** Percentage of occupational groups for each country and total.

	**Brazil (*n* = 524)**	**Portugal (*n* = 522)**	**Total (*N* = 1,046)**
**OCCUPATIONAL GROUPS**			
Armed Forces Occupations	1.32	4.78	3.07
Managers	16.56	9.35	12.92
Professionals	33.77	51.74	42.83
Technicians and Associate Professionals	9.27	13.48	11.39
Clerical Support Workers	28.26	9.35	18.73
Services and Sales Workers	6.18	6.30	6.24
Skilled Agricultural, Forestry and Fishery Workers	–	–	–
Craft and Related Trades Workers	1.99	2.39	2.19
Plant and Machine Operators and Assemblers	0.88	0.65	0.77
Elementary Occupations	1.77	1.96	1.86
**ACADEMIC LEVEL**			
PhD	5.18	5.26	5.22
Master	9.07	38.11	23.77
Post-graduation (not master neither PhD)	23.97	9.90	16.84
Graduation	34.77	29.26	31.98
Unfinished graduation	12.96	4.84	8.85
High school, vocational education or less	14.05	12.63	13.34

### Measures

To assess work engagement, we used the Utrecht Work Engagement Scale short version UWES-9 (Schaufeli and Bakker, 2003), developing a new version for the Portuguese language transculturally adapted both for Brazil and Portugal (Table 3). The UWES-9 is a self-report scale scored on a 7-point rating scale (0 = never; 6 = always) with three questions each about the three dimensions of vigor, dedication, and absorption. The UWES-9 was chosen because previous research with the UWES-9 has shown validity evidence across different countries such as Japan, Spain, Norway and China (Shimazu et al., 2008; Nerstad et al., 2010; Fong and Ng, 2012).

**Table 3:** UWES-9 original and Portuguese versions.

**Item**	**Original UWES–9 (Schaufeli and Bakker**, **2003****)**							**Portuguese (Brazil and Portugal) version of UWES-9**						
	Never	Almost never	Rarely	Sometimes	Often	Very often	Always	Nunca	Quase nunca	Às vezes	Regularmente	Frequentemente	Quase sempre	Sempre
	0	1	2	3	4	5	6	0	1	2	3	4	5	6
	Never	A few times a year or less	Once a montd or less	A few times a montd	Once a week	A few times a week	Every day	Nenhuma vez	Algumas vezes por ano	Uma vez ou menos por mês	Algumas vezes por mês	Uma vez por semana	Algumas vezes por semana	Todos os dias
	**Vigor**							**Vigor**						
1	At my work, I feel bursting with energy							Sinto-me cheio de energia no meu trabalho						
2	At my job, I feel strong and vigorous							Sinto-me com força e vigor no meu trabalho						
5	When I get up in the morning, I feel like going to work							Quando me levanto pela manhã, tenho vontade de ir trabalhar						
	**Dedication**							**Dedicação**						
3	I am enthusiastic about my job							Sou uma pessoa entusiasmada com o meu trabalho						
4	My job inspires me							O meu trabalho inspira-me						
7	I am proud on the work that I do							Tenho orgulho no trabalho que realizo						
	**Absorption**							**Absorção**						
6	I feel happy when I am working intensely							Sinto-me feliz quando estou intensamente envolvido no trabalho						
8	I am immersed in my work							Fico absorvido com o meu trabalho						
9	I get carried away when I'm working							Sinto-me tão empolgado, que me deixo levar quando estou a trabalhar						

Thus, to develop this Portuguese version, we used UWES' original manual (Schaufeli and Bakker, 2003) and we followed the *International Test Commission guidelines for translating and adapting tests* (International Test Commission, 2017) adapting the items to the Portuguese language per the Orthographic Agreement signed by both Portugal and Brazil in 2009. We discussed the items with Portuguese and Brazilian specialists on psychology and methodology, to create a version of the items that gathered the consensus of the specialists regarding the cultural, semantic and idiomatic equivalence in both countries. Finally, we did a small pilot test with 15 workers from each country, who didn't suggest any modifications and understood the adapted UWES-9 items. The final single version (for both countries) had no other changes besides the translation to adapt words according to each country's specificities.

### Procedures

Data were gathered from 2014 to 2017, simultaneously in both countries, in an effort to respect each institution's authorization and to have a larger sample, since web surveys present low response rates (Massey and Tourangeau, 2013). The participants filled a self-report psychometric instrument (UWES-9) and a brief sociodemographic questionnaire, both of which were available online using LimeSurvey software (Limesurvey GmbH, 2017) running on the website of each faculty in each country. Using several contact persons, researchers invited, in person or via e-mail, 260 Portuguese and 280 Brazilian companies to disseminate the study and ask for voluntary participation among nearby 1,500 workers of each country. Nearly 35% of the disseminated questionnaires were completed in both countries. Before they completed the survey, some information about the study was presented, explaining the aims and explaining that it was a research study where the company would not access individual data, and that companies simply helped the researchers disseminating the study. Informed consent was obtained online from all participants. The online survey presented, to begin with, information about the study's aims (and an institutional mail to answer doubts if the participant wanted), and also the Consent Form: “*I agree to participate in this research study. I understand the purpose and nature of this study and I'm participating voluntarily. My answers in this study will remain confidential. I can withdraw my participation at any time*.” If the person agreed to participate, then he/she clicked “Yes” and was prompted to begin completing the survey. Upon completing the survey, participants were presented the sentence: “*Thank you for your participation. It is not possible to identify your questions individually and the analysis will be performed as aggregated data by university researchers*,” aiming to reinforce the confidential nature of data.

To allow comparative studies, the same procedures were used in both countries. The study was approved by the Ethics Committee of the University of Porto, Portugal, and the University of São Paulo, Brazil, and followed the usual rules for online surveys, namely no access of participants' companies to individual results, and no direct contact between participants and researchers (a few used the email to clarify some details about the access to individual data, but it is not possible to identify if they participated in the study).

### Data analysis

Aiming to confirm the original structure of the instrument, a confirmatory factor analysis (CFA) was conducted to verify if the proposed 3-factor structure presented an adequate fit to the study sample. We used as the goodness-of-fit indices the TLI (Tucker Lewis Index), NFI (Normed Fit Index), χ^2^/df (ratio chi-square and degrees of freedom), CFI (comparative fit index) and the RMSEA (root mean square error of approximation). The fit of the model was considered good for χ^2^/df smaller than 5, CFI, NFI and TLI values above 0.95 and RMSEA values below 0.08 (Hoyle, 1995; Boomsma, 2000; McDonald and Ho, 2002; Byrne, 2016). All statistical analyses were performed with R (R Core Team, 2017) and Rstudio (RStudio Team, 2017). The descriptive statistics were obtained with the skimr package (Arino de la Rubia et al., 2017). The CFA analysis was conducted with the lavaan package (Rosseel, 2012) using the Weighted Least Squares Means and Variances (WLSMV) estimation method.

To analyze the convergent validity evidence, the average variance extracted (AVE) was estimated as described in Fornell and Larcker (1981) and Marôco (2014). Values of AVE ≥ 0.5 were considered indicative of the constructs' convergent validity evidence of the UWES' factors (Hair et al., 2009).

For discriminant validity evidence, to check if the items that represent a dimension were not strongly correlated with other dimensions (Marôco, 2014), discriminant validity evidence was checked (Fornell and Larcker, 1981; Marôco, 2014): for two factors *x* and *y*, if AVE*x* and AVE*y* ≥ $\rho_{xy}^{2}$ (squared correlation between the factors *x* and *y*) there is discriminant validity evidence.

The reliability of the scores was assessed with estimates of internal consistency, the Composite Reliability (CR), ordinal α (Oliden and Zumbo, 2008) and ω (Raykov, 2001); higher values were indicative of better internal consistency results.

The reliability estimates and measurement invariance for the first-order three-factor model taking in account the categorical items with theta-parameterization (Millsap and Yun-Tein, 2004) were calculated with the semTools package (SemTools Contributors, 2016). Measurement invariance for the second-order model was assessed with the lavaan package (Rosseel, 2012), comparing a group of seven different models based on the recommendations of Millsap and Yun-Tein (2004) and on the second-order models' invariance specificities (Chen et al., 2005): (a) configural invariance, (b) first-order factor loadings, (c) second-order factor loadings, (d) thresholds of measured variables, (e) intercepts of first-order factors, (f) disturbances of first-order factors, and (g) residual variances of observed variables. The percentiles were calculated using the doBy package (Højsgaard and Halekoh, 2016). The comparisons of the raw levels of the UWES' factors between countries' groups were addressed using a *t*-student test for independent groups using the stats package (R Core Team, 2017). The effect sizes (Cohen's *d*) were calculated using the lsr package (Navarro, 2015).

## Results

### Validity evidence based on internal structure

#### Dimensionality

##### Items' distributional properties

Summary measures, skewness (*sk*), kurtosis (*ku*) and a histogram for each of the nine items are presented (Table 4) and were used to judge distributional properties and psychometric sensitivity. Absolute values of *ku* smaller than 7 and *sk* smaller than 3 were considered as indicative of no strong deviations from the normal distribution (Finney and DiStefano, 2013). The Mardia's Multivariate Kurtosis for the nine items of UWES was 90.36; *p* < 0.001. All possible answer values for each item were also present, and no outliers were deleted. These items' distributional properties are indicative of appropriate psychometric sensitivity, as it would be expected that these items would follow an approximate normal distribution in the population under study. Despite these univariate and multivariate normality indicators, the WLSMV estimator was used, taking into consideration the ordinal level of measurement of the items.

**Table 4:** UWES' items descriptive statistics.

**UWES-9 items**	***Mean***	***SD***	**Minimum**	**Maximum**	**Skewness**	**Kurtosis**	**Histogram**
UWES1^V^	4.14	1.38	0	6	−0.73	−0.09	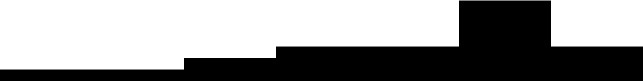
UWES2^V^	4.14	1.42	0	6	−0.77	−0.04	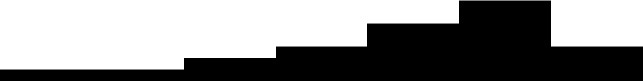
UWES3^D^	4.31	1.54	0	6	−0.84	−0.15	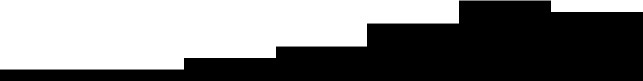
UWES4^D^	4.05	1.72	0	6	−0.66	−0.59	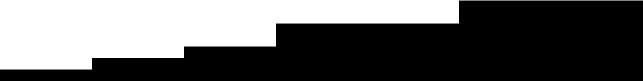
UWES5^V^	3.74	1.78	0	6	−0.53	−0.77	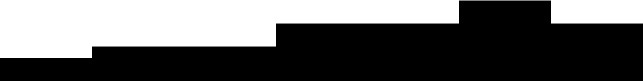
UWES6^A^	4.54	1.54	0	6	−1.13	0.56	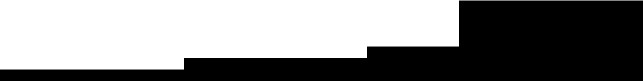
UWES7^D^	4.72	1.53	0	6	−1.37	1.26	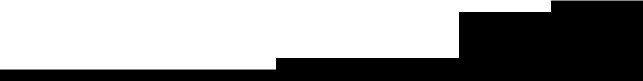
UWES8^A^	4.44	1.51	0	6	−1.04	0.45	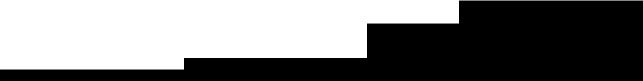
UWES9^A^	3.98	1.71	0	6	−0.70	−0.40	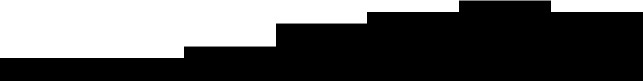

*V, Vigor items; D, Dedication items; A, Absorption items*.

##### Factor related validity evidence

**Figure 1:**
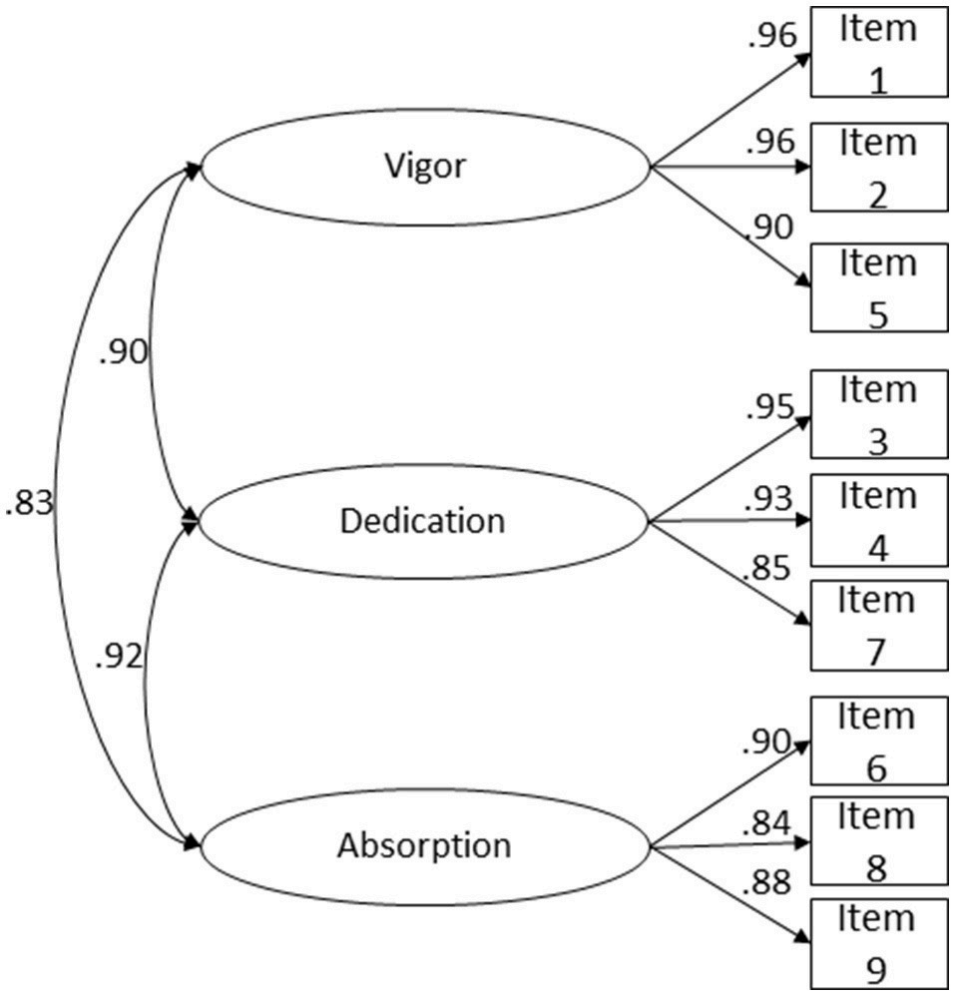
UWES-9 tri-factor structure fit to a combined sample of Portuguese (*n* = 520) and Brazilian Workers (*n* = 524). Correlations between latent variables, and factor loadings for each item are shown $\chi_{\left(24\right)}^{2}$ = 408.520, *p* < 0.001, *N* = 1,046, *CFI* = 0.998, *NFI* = 0.998, *TLI* = 0.997, *RMSEA* = 0.124, *P(RMSEA* ≤ 0.05) < 0.001, 90% CI (0.113; 0.135).

The 3-factor model fit to the data was acceptable (Figure 1), since CFI, NFI, and TLI values were above 0.95, but RMSEA values were above 0.10, while the factor weight of all items was above 0.84.

##### Convergent validity evidence

The AVE was good, for Vigor (0.89), for Dedication (0.83) and Absorption (0.76). These results suggest good convergent validity evidence for the UWES-9 and demonstrated that items contained within each factor are related to each other.

##### Discriminant validity evidence

Regarding the discriminant validity evidence, *AVE*_vigor_ = 0.89 and *AVE*_dedication_ = 0.83 were bigger than $r_{\text{VD}}^{2}$ = 0.81, the *AVE*_absorption_ = 0.76 and *AVE*_dedication_ = 0.83 were both smaller than $r_{\text{AD}}^{2}=$ 0.85, and the *AVE*_vigor_ = 0.89 and and the *AVE*_absorption_ = 0.76 were both bigger than $r_{\text{VA}}^{2}=$ 0.69. The discriminant validity evidence was good between Vigor and Absorption and between Vigor and Dedication, and bad between Dedication and Absorption. These findings showed that some factors are strongly related to each other.

##### Second-order construct

Second-order models are potentially applicable when the first-order factors are highly correlated with each other, and when there is a higher order factor that possibly is responsible for the relations between the first-order factors (Chen et al., 2005; Marôco, 2014).

**Figure 2:**
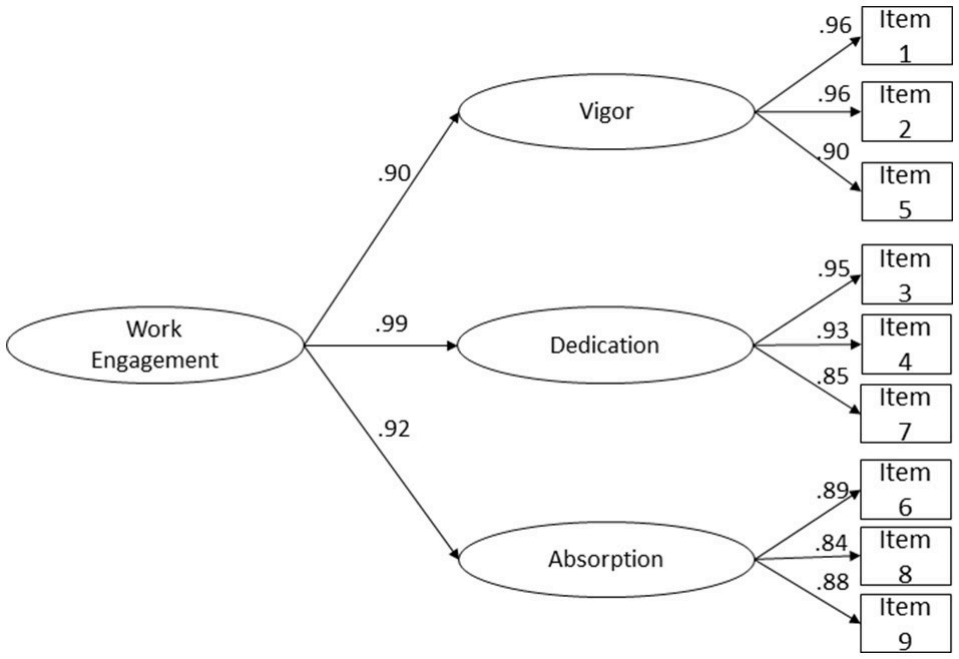
UWES-9 second-order latent factor structure fit a combined sample of Portuguese (*n* = 522) and Brazilian Workers (*n* = 524). Second-order latent loadings for each factor, and factor loadings for each item are shown $\chi_{\left(25\right)}^{2}$ = 409.919, *p* < 0.001, *N* = 1,046, *CFI* = 0.998, *NFI* = 0.998, *TLI* = 0.997, *RMSEA* = 0.121, *P(RMSEA* ≤ 0.05) < 0.001, 90% CI (0.111; 0.132).

Considering the high correlations between *Vigor, Dedication* and *Absorption* and the lack of discriminant validity evidence between the Dedication and Absorption factors, we added a second-order factor that we named *Work Engagement*. The goodness-of-fit of the second-order model was considered acceptable (Figure 2), and the loadings of *Work Engagement* on its first-order factors were quite high and statistically significant (*p* < 0.001): 0.88 for *Vigor*, 0.98 for *Dedication* and 0.94 for *Absorption*, suggesting that the second-order factor is equally defined by the first-order dimensions of the work engagement scale.

Considering previous analysis and high correlations between the factors, we propose that the UWES-9 can be used to define work engagement as a second-order factor (Chen et al., 2005). This model was supported by the data, showing better goodness-of-fit indices than the three-factor (one-order) solution. This indicates that in addition to the three UWES subdimensions, there is also a more general domain-specific work engagement. With this solution, we keep aligned with the theoretical three-factor division of UWES (Schaufeli and Bakker, 2003), but also show that work engagement can be a second-order dimension.

#### Reliability of the scores: internal consistency evidence

Regarding internal consistency (Table 5), ordinal Cronbach's α for the total sample was 0.95, suggesting very good internal consistency evidence. Additionally, we presented other ordinal reliability estimates (CR and ω) to allow future comparisons with other studies. Those other estimates also presented good internal consistency evidence.

**Table 5:** Internal consistency of UWES dimensions.

**UWES-9 dimension**	**Ordinalα_Total sample_**	**Ordinalω_Total sample_**	**CR _Total sample_**
Vigor	0.93	0.94	0.96
Dedication	0.93	0.92	0.94
Absorption	0.90	0.88	0.91
Total	0.96	0.97	–

The proportion of the second-order factor explaining the variance at first-order factors' level (ω_L2_) was 0.96, the proportion of the second-order factor explaining the total score (ω_L1_) was 0.93, and the proportion of observed variance explained by the second-order factor after controlling for the uniqueness from the first-order factor (ω_partialL1_) was 0.97. Thus, the internal consistency of the higher-order construct was very good.

#### Measurement invariance

To detect whether the same original three-factor model holds in each country (Table 6), a group of nested models with indications of equivalence is needed (Marôco, 2014). This should be done considering the ordinal nature of the scale. Thus, we tested measurement invariance for categorical items with theta-parameterization. Full uniqueness measurement invariance was supported by the Cheung and Rensvold (2002) criterion (ΔCFI < 0.01), and also partial scalar invariance by the (more) restrictive Δχ^2^ criterion (Satorra and Bentler, 2001); the test comparing the fit of the constrained vs. free models is not statistically significant. Results supported the structural invariance between Portugal and Brazil.

**Table 6:** Model comparison between Portugal and Brazil.

**Model invariance**	**χ^2^**	***df***	**χ^2^/*df***	***CFI* scaled**	**Δχ^2^**	**Δ*CFI* scaled**
Configural (factor structure)	455.06	48	9.48	0.985	–	–
Metric (loadings)	457.74	54	8.48	0.985	4.88^ns^	0.000
Scalar (thresholds)	500.83	96	5.22	0.987	66.62^**^	0.002
Full uniqueness (residuals)	609.22	105	5.80	0.986	100.97^***^	0.002
Latent means	619.79	108	5.74	0.993	0.439^ns^	0.008

ns p > 0.05;

** p < 0.01;

*** *p < 0.001*.

Regarding the UWES-9 second-order three-factor structure, full uniqueness measurement invariance was supported by the Cheung and Rensvold (2002) criterion (ΔCFI < 0.01), and the Δχ^2^ (Satorra and Bentler, 2001) supported (partial) scalar invariance for the models comparison between countries (Table 7).

**Table 7:** UWES-9 second-order three-factor latent model comparison between countries.

**Model**	**χ^2^**	***df***	**χ^2^/*df***	***CFI* scaled**	**Δχ^2^**	**Δ*CFI* scaled**
Configural (factor structure)	455.06	48	9.48	0.985	–	–
First-order loadings invariance	457.74	54	8.48	0.985	3.58^ns^	0.000
Second-order loadings invariance	458.71	56	8.19	0.986	1.53^ns^	0.001
Thresholds of measured variables	501.94	98	5.12	0.988	33.33^*^	0.002
Intercepts of first-order factors invariance	533.43	101	5.28	0.994	2.50^ns^	0.006
Disturbances of first-order factors invariance	588.54	107	5.50	0.994	43.57^***^	0.000
Residual variances of observed variables invariance	733.89	116	6.33	0.993	73.06^***^	0.001

ns p > 0.05;

* p < 0.05;

*** *p < 0.001*.

### Dimensions' comparisons

Finally, since we had measurement invariance between samples, we performed a comparative analysis of the UWES dimensions between the two countries (Table 8). There were no statistically significant differences on the UWES dimensions between Portuguese and Brazilian workers.

**Table 8:** Comparative analysis between countries (means, *SD* and percentiles).

**UWES-9 dimension**	**Brazil (*****n*** = **524)**		**Portugal (*****n*** = **520)**		***t*-student**	***df***	***p***	**Cohen's *d***	**Brazil**			**Portugal**		
	***M***	***SD***	***M***	***SD***					**25**	**50**	**75**	**25**	**50**	**75**
Vigor	4.02	1.46	4.00	1.35	0.21	1,044	0.83	0.01	3.00	4.00	5.00	3.00	4.33	5.00
Dedication	4.39	1.52	4.34	1.41	0.56	1,044	0.57	0.03	3.33	5.00	5.67	3.33	4.67	5.33
Absorption	4.33	1.46	4.31	1.4	0.33	1,044	0.74	0.02	3.33	4.67	5.67	3.67	4.67	5.33

## Discussion

Our results confirmed four of the five hypotheses. The UWES-9 tri-factorial first-order model showed validity evidence that allows its use among workers from Brazil and Portugal (H1). This result is in line with several studies showing that the UWES-9 three-factor first-order model has acceptable (at least) psychometric properties (Villotti et al., 2014; Flores Jiménez et al., 2015; Vazquez et al., 2015; Sinval et al., 2018), although other studies presented better fit with other alternative model structures (de Bruin and Henn, 2013; Ho Kim et al., 2017).

Our results also revealed that this version of the UWES-9 presented measurement invariance for Portugal and Brazil, which allows its use in comparative studies between these countries and confirms the second hypothesis (H2). In fact, measurement invariance has also been found between other countries: samples of white-collar workers from the Netherlands and Italy presented metric invariance (Balducci et al., 2010); UWES-15 version between Greek and Dutch workers (Xanthopoulou et al., 2012); the student version (UWES-S) presented invariance only for the absorption dimension among samples of students from Portugal, Spain, and the Netherlands (Schaufeli et al., 2002a). Our findings support the need for more studies to test measurement invariance across countries (Schaufeli et al., 2006). Other studies demonstrated factorial invariance for members of the same occupational group between different countries or ethnic groups (Storm and Rothmann, 2003), which, in turn, makes UWES' measurement invariance more likely. Additionally, no factorial invariance across samples from various countries of different continents was found in the study of Schaufeli et al. (2006).

Regarding the third hypothesis, the second-order latent factor *work engagement* was proposed following concerns with the high correlations between the three latent factors of the UWES-9 found in various studies (Schaufeli et al., 2002b; Schaufeli and Bakker, 2003). Our results confirmed H3, and this kind of approach isn't a novelty in the sense that others tried this with two first-order latent factors, vigor and dedication (Alok, 2013) and with three first-order latent factors (Sinval et al., 2018). However, in the first study a one-factor model presented better fit, and in the second study the second-order model had a better fit than the first-order one in a Portuguese sample of rescue workers. Moreover, no studies with second-order UWES' models were found for samples of workers from Brazil.

Concerning the measurement invariance of this second-order model (H4), our study obtained a novel finding, since we found measurement invariance for the level of residual variances of observed variables across samples of workers from various occupational groups of both countries. Partial metric invariance was found before (Sinval et al., 2018) for a second-order model across different occupations (firefighters, nurses, and police officers), although this is the first study that presents full measurement invariance across different countries for a second-order model of the UWES-9, which is particularly interesting given the fact that these two countries are from different continents. The vast majority of the studies on work engagement were conducted in countries from North America and Western Europe (Hu et al., 2014), but our study included a South America country that can be compared with a European country regarding *work engagement*.

The dimensions of UWES-9 didn't present statistically significant different levels between Brazil and Portugal, rejecting H5, formulated based on socio-cultural differences between these countries. However, no other studies comparing Portugal and Brazil in terms of *work engagement* with this instrument were found, since other studies measured the three UWES dimensions in Portugal and Brazil individually in various occupations (Marques-Pinto and Picado, 2011; Vazquez et al., 2015), using different versions of the instrument; thus, their findings could not be directly compared with ours. This enforces the utility of using our version of the UWES-9 to establish larger comparisons with rigor between two brother countries. Maybe socio-cultural differences are not so strong between Portugal and Brazil, or since data were collected during the world economic crisis, the workers are thankful to have a job (work engagement levels are moderate to high) and work engagement levels are not affected by the country. Having now a unique instrument in the Portuguese language, further studies can be developed, since cross-cultural studies between Portugal-Brazil are not that frequent.

After presenting a revision of the international versions of Utrecht Work Engagement Scale, this paper aimed to investigate the validity evidence related to the scale dimensionality of the Portuguese version of the UWES-9 developed simultaneously for Brazil and Portugal, namely, dimensionality, measurement invariance between Brazil and Portugal, and reliability of the scores. Our version showed good convergent validity evidence and acceptable discriminant validity evidence, and measurement invariance evidence for both the first- and second-order models for use in the Portuguese vs. Brazilian cultural context. The goodness-of-fit indices were good/acceptable for the UWES nine item version. This solution is corroborated by some international studies and may have been found because work engagement, as measured by the UWES, is a construct with high correlations between factors (Mauno et al., 2007; Hakanen et al., 2008; Weigl et al., 2010; Federici and Skaalvik, 2011; Chughtai and Buckley, 2013; Agarwal, 2014). In fact, a meta-analytic study found very high correlations between UWES factors: 0.95 between vigor and absorption, 0.90 between dedication and absorption and 0.88 between vigor and dedication (Christian and Slaughter, 2007). We suggested that a second-order latent factor might account for such intercorrelations, and in our study it presented an acceptable fit. It is very common that when conducting confirmatory factor analysis, a three-factor solution may not be clear, but still fits as a possible model to interpret the work engagement results (Schaufeli and Bakker, 2010).

Previous research showed (see Table 1) that the UWES-9 three-factor solution was invariant between Italy and Dutch white-collar workers samples (Balducci et al., 2010), but with two versions of the instrument, one in each language. Although measurement invariance wasn't obtained in a study between 10 countries with the UWES-9 (Schaufeli et al., 2006), the UWES showed invariance in South African police officers of different racial groups (Storm and Rothmann, 2003). Also, for different Japanese occupations, the UWES-9 (one factor) showed measurement invariance (Shimazu et al., 2008). For a Dutch, Spanish and Portuguese student sample, the UWES-S (student survey) showed invariance only for the Absorption factor across the three countries; three-factor structure invariance wasn't obtained (Schaufeli et al., 2002a). However, in this study, with a sample of Portuguese and Brazilian workers, the results revealed measurement invariance, allowing comparisons of means between these two countries using the same UWES-9 version. Our results are in line with those obtained by Seppälä et al. (2009), who analyzed five different studies (*N* = 9,404), including a three-year longitudinal study (*n* = 2,555) among different occupational samples, concluding that vigor, dedication, and absorption presented a correlated three-factor structure, and although the structure of the UWES-17 did not remain the same across the samples and time, the structure of the UWES-9 remained relatively unchanged. Although Schaufeli et al. (2006) argued that measurement invariance is less likely to be obtained between different occupational groups, Hernandez-Vargas et al. (2016) found invariance across two Mexican samples, and our results showed measurement invariance across countries with samples containing different occupational groups. Moreover, the UWES-9 showed different means and percentiles for each country, with Portugal presenting higher values for vigor and absorption, which could be explained by different cultural values (Hofstede et al., 2010) or occupational activities (see Table 2) related primarily to mental demands instead of physical demands, and thus increasing absorption levels. Cross-cultural analysis from Hu et al. (2014) compared work engagement across East Asia (China and Japan) and Western Europe (Finland, Netherlands, and Spain), concluding that European employees were more engaged than Asian employees. Recently, Schaufeli (2017) argued that work engagement seems to be positively related to nations' economic activity, and also with lower work centrality (thus valorizing leisure over work), strong democracy, high integrity, low corruption, gender inequality, and individualistic culture.

## Conclusion

This Portuguese transcultural UWES-9 version has good construct validity evidence, and its use can be recommended in future research, namely to perform comparative studies between Portuguese-speaking countries or groups, to analyze work engagement's association with other recent constructs, such as job crafting as a protector from job boredom (Harju et al., 2016), the relationship of work engagement with productivity or person-job fit (De Beer et al., 2016; Fuller and Shikaloff, 2017), or occupational differences in work engagement (Innstrand, 2016) or to perform longitudinal studies (Seppälä et al., 2009). Additionally, we will be able to understand if these different groups/countries can differently perceive work engagement between them according to cultural and organizational values (Hofstede et al., 2010). Also, multi-cultural workforces that are increasing in each country (OECD, 2016) challenge researchers to pursue new research topics such as psychological assessment and interventions with multicultural populations (Dana, 2000; López, 2000). A transculturally valid UWES scale can aid research efforts to study migrant flows and employees' well-being and productivity between Portugal and Brazil.

The study has some limitations, namely the convenience sample and the fact that only those with a professional occupation participated. Additionally, the samples were dependent on voluntary participation, which can elicit some bias or reflect the healthy worker myth, which means that those who are satisfied participate more in research studies, while the most disengaged are too stressed to collaborate, according to Shah (2009). In the future, it will be important to also evaluate workers' situations as migrant or native, since it can affect their relationship with institutions and colleagues, thus influencing their work engagement. Moreover, other structures can be tested in the future, such as two latent factors (Gilchrist et al., 2013; Sulaiman and Zahoni, 2016).

Regarding theoretical implications, considering previous analysis and high correlations between the factors, we propose that the UWES-9 can be used to define work engagement as a second-order factor. This model was supported by the data, showing better goodness-of-fit indices than the three-factor (one-order) solution. This indicates that in addition to the three UWES sub-dimensions, there is also a more general domain-specific work engagement. With this solution, we keep aligned with the theoretical three-factor division of the UWES, but also show that work engagement can be a second-order construct. This is one of the main novelties of our study.

As practical implications, the UWES-9 transcultural version presented acceptable psychometric properties and invariance between Portugal and Brazil, allowing cross-cultural studies between these countries. Recent international workforce flows present a challenge for human resource management, since migrants are used to increase productivity, but they can have different perceptions about the organization where they are employees (Pocnet et al., 2015; Wojczewski et al., 2015; Le et al., 2016). Migrant workers' work engagement with labor market has already attracted researchers' interest (Samaluk, 2016) and UWES seems to be an important measure to understand employees' work engagement in the organizations, allowing human resources departments to better adapt their practices to workforces, especially when the workers are migrants. Moreover, as Schaufeli (2017) recently argued, work engagement may not only be studied at the individual, psychological level, but also at the collective and national level as it relates in meaningful ways with various economic and sociocultural indicators, helping organizations to increase their productivity due to engaged workers who present high performance and job satisfaction (Christian et al., 2011).

## Ethics statement

This study was carried out in accordance with the recommendations of the Ethics guidelines, of the FPCEUP and FFCLRP-USP Ethics Committees with written informed consent from all subjects. All subjects gave written informed consent in accordance with the Declaration of Helsinki. The protocol was approved by the FPCEUP and FFCLRP-USP Ethics Committees.

## Author contributions

All authors of this research paper have directly participated in the planning, execution, or analysis of this study. More specifically, JS wrote the paper, and with JM performed all statistical analysis and its discussion. JS and SP discussed cross-cultural topics, and JS and CQ discussed theoretical framework.

### Conflict of interest statement

The authors declare that the research was conducted in the absence of any commercial or financial relationships that could be construed as a potential conflict of interest.
